# Fixed-Dose Ultrasound-Assisted Catheter-Directed Thrombolysis for Acute Pulmonary Embolism Associated with COVID-19

**DOI:** 10.3390/v14081606

**Published:** 2022-07-22

**Authors:** Davide Voci, Stéphanie Zbinden, Evy Micieli, Nils Kucher, Stefano Barco

**Affiliations:** 1 Department of Angiology, University Hospital Zurich, 8091 Zurich, Switzerland; davide.voci@usz.ch (D.V.); stephanie.zbinden@usz.ch (S.Z.); evy.micieli@usz.ch (E.M.); nils.kucher@usz.ch (N.K.); 2 Center for Thrombosis and Hemostasis, Johannes Gutenberg University Mainz, 55131 Mainz, Germany

**Keywords:** pulmonary embolism, thrombolysis, venous thromboembolism, COVID-19, SARS-CoV2

## Abstract

**Background**. Fixed-dose ultrasound-assisted catheter-directed thrombolysis (USAT) rapidly improves hemodynamic parameters and reverses right ventricular dysfunction caused by acute pulmonary embolism (PE). The effectiveness of USAT for acute PE associated with coronavirus disease 2019 (COVID-19) is unknown. **Methods and results**. The study population of this cohort study consisted of 36 patients with an intermediate-high- or high-risk acute PE treated with a fixed low-dose USAT protocol (r-tPA 10–20 mg/15 h). Of these, 9 patients tested positive for COVID-19 and were age–sex-matched to 27 patients without COVID-19. The USAT protocol included, beyond the infusion of recombinant tissue plasminogen activator, anti-Xa-activity-adjusted unfractionated heparin therapy (target 0.3–0.7 U/mL). The study outcomes were the invasively measured mean pulmonary arterial pressure (mPAP) before and at completion of USAT, and the National Early Warning Score (NEWS), according to which more points indicate more severe hemodynamic impairment. Twenty-four (66.7%) patients were men; the mean age was 67 ± 14 years. Mean ± standard deviation mPAP decreased from 32.3 ± 8.3 to 22.4 ± 7.0 mmHg among COVID-19 patients and from 35.4 ± 9.7 to 24.6 ± 7.0 mmHg among unexposed, with no difference in the relative improvement between groups (*p* = 0.84). Within 12 h of USAT start, the median NEWS decreased from six (Q1–Q3: 4–8) to three (Q1–Q3: 2–4) points among COVID-19 patients and from four (Q1–Q3: 2–6) to two (Q1–Q3: 2–3) points among unexposed (*p* = 0.29). One COVID-19 patient died due to COVID-19-related complications 14 days after acute PE. No major bleeding events occurred. **Conclusions**. Among patients with COVID-19-associated acute PE, mPAP rapidly decreased during USAT with a concomitant progressive improvement of the NEWS. The magnitude of mPAP reduction was similar in patients with and without COVID-19.

## 1. Introduction

The initial management of acute pulmonary embolism (PE) is conditional on hemodynamic impairment and patient clinical characteristics. Hemodynamically unstable (high-risk) PE patients are characterized by an immediate risk of death. Systemic thrombolysis is indicated to swiftly relieve the pulmonary artery obstruction [1,2,3]. Among patients who appear to be hemodynamically stable but present with severe right ventricular dysfunction and positive troponin (intermediate-high risk PE patients), systemic thrombolysis is not routinely recommended, as the associated risk of major bleeding outweighs the clinical benefits [4,5].

Prior interventional studies showed that (ultrasound-assisted) catheter-directed thrombolysis (USAT) can improve echocardiographic and hemodynamic parameters among intermediate- and high-risk acute PE patients [6,7,8]. However, the lack of evidence from randomized controlled trials adopting “hard” clinical outcomes prevent USAT being recommended as a first-line treatment [9]. USAT represents a rescue therapy for patients with hemodynamic deterioration, or if systemic thrombolysis is contraindicated or did not lead to a remarkable hemodynamic improvement [5].

Pulmonary embolism is a frequent complication of COVID-19, particularly among hospitalized patients [10]. The in-hospital incidence of bleeding complications is not negligible, particularly in patients with multiorgan failure, multiple vascular access, and thrombocytopenia [11]. The prognosis of patients with COVID-19-associated PE is worse than that of patients without COVID-19 [12]. It has been postulated that thrombolytic agents might improve survival among COVID-19, even in the absence of a hemodynamically unstable PE [13]. It is unknown whether USAT can normalize the hemodynamic parameters in patients with both COVID-19 and a severe right ventricular dysfunction based on available data coming from isolated case reports [14,15,16].

In this cohort study, we studied the effectiveness of USAT for COVID-19-associated acute PE in terms of hemodynamic parameters. We compared it to age–sex-matched patients without COVID-19.

## 2. Materials and Methods

This study was conducted as an observational retrospective single-center cohort study to investigate the effectiveness of USAT in acute PE patients with and without COVID-19. We performed an initial screening of patients with intermediate-high or high-risk acute PE who were treated at the Department of Angiology of the University Hospital Zurich with an ultrasound-assisted catheter-directed system (EKOS^®^, Boston Scientific, Marlborough, MA, USA) between September 2018 and May 2021. This tertiary academic hospital is one of the two national reference centers focusing on endovascular therapies of acute PE. The Department of Angiology leads the local Pulmonary Embolism Response Team (PERT). USAT is primarily considered for rescue thrombolysis or for the acute management of patients presenting with right ventricular dysfunction (right-to-left ventricular [RV/LV] ratio > 1.0), positive troponin, centrally located emboli in at least one main or lobar pulmonary artery, and an estimated high risk of hemodynamic collapse based on clinical parameters, such as worsening tachycardia, blood pressure, or oxygenation. RV/LV ratio is measured with contrast-enhanced computed tomography (CT) or transthoracic echocardiography.

According to the protocol used in the Ultrasound Accelerated Thrombolysis of PE (ULTIMA) trial, [6] USAT was administered as a continuous infusion of recombinant tissue plasminogen activator (rtPA) at 1 mg/h and saline coolant at 35 mL/h per catheter over 5 h for a total of 5 mg. Thereafter, the infusion rate of rtPA was reduced to 0.5 mg/h per catheter (if two catheters are put in place) or to 1 mg/h (if one catheter is used) and administered for 10 h. Thrombolysis duration was 15 h in all patients. Consequently, the maximum rtPA dose was 20 mg for patients with bilateral PE and two catheters placed, whereas a 10 mg total dosage was used for patients with unilateral PE and only one catheter placed [6]. During USAT, patients were monitored at the intermediate care unit or, in exceptional cases, at the intensive care unit.

All patients received standard treatment with unfractionated heparin (UFH). An initial bolus (80 units/kg) was given upon clinical suspicion/PE diagnosis and, thereafter, anti-factor-Xa-adjusted UFH infusion over at least 24 h. Fibrinogen and anti-factor-Xa levels were monitored every 4–6 h during USAT with a consequent reduction of the alteplase dose if fibrinogen dropped more than 50% from baseline or below 1 g/L or if the anti-faxtor-Xa was outside the institutional reference range of 0.3–0.7 IU/mL [17]. For this analysis, we calculated the time in therapeutic range for UFH by adapting the Rosendaal’s method of interpolated values [18]. A switch to direct oral anticoagulants and transfer to the regular ward was permitted following removal of the catheters.

Prior to placement of the EKOS^®^ catheters, invasive mPAP was measured from the main pulmonary stem using a 5-French pigtail or multipurpose diagnostic catheter. After completion of USAT, invasive mPAP was measured in the monitor unit via the EKOS^®^ catheter after removal of the microsonic device and by pulling back the catheter approximately 10 cm until an undamped pressure curve was observed.

The following outcomes have been pre-specified in the study protocol approved by the ethical committee before the start of data collection: (i) mean pulmonary arterial pressure (mPAP) measured invasively before and immediately after USAT and NEWS during the first 12 h of USAT; (ii) hemodynamic decompensation and death within 30 days of acute PE; (iii) other complications (PE recurrence, major bleeding, stroke, device-related complications) within 30 days of acute PE. For NEWS, an assessment after 12 h from USAT start was decided, as the values measured at the end of USAT may have been affected by procedures related to catheter removal.

Follow-up data were obtained from routinely collected data available in our online medical charts, notably hemodynamic and cardiopulmonary parameters during initial hospitalization at the intermediate or intensive care unit, including heart rate, respiratory rate, and oxygen saturation. The National Early Warning Score (NEWS), as a proxy of the general hemodynamic status, was retrospectively calculated for each patient, as its components (respiratory rate, oxygen saturation, supplemental oxygen, body temperature, systolic blood pressure, heart rate, level of consciousness) are hourly monitored in all patients during intermediate care unit stay. A general consent for the use of personal data for research purposes was introduced at our institution in 2017 and was obtained from the subjects (or their legally authorized representative). The local Ethical Commission approved the study protocol on 25 March 2021 (BASEC ID 2021-00579). The study complies with the Declaration of Helsinki. Individual patient data are available upon request after approval of the local ethical commission. The study received unrestricted funding from the manufacturer of the EKOS^®^ catheter system (Boston Scientific, Marlborough, MA, USA).

Descriptive analyses of the baseline characteristics used counts and percentages for categorical data, whereas continuous data were expressed as mean and standard deviation (SD) or as medians and quartiles 1–3 (Q1-Q3). Comparisons between groups for continuous variables were conducted with paired or unpaired parametric or nonparametric tests, as appropriate and specified for each analysis. The changes in NEWS were calculated as absolute values, as well as percentage of change of the initial value. IBM SPSS version 26.0 (IBM Corp., Armonk, NY, USA) was used for statistical analysis.

## 3. Results

### 3.1. Characteristics of the Study Population

We screened 142 patients with acute PE who received USAT at the University Hospital Zurich between September 2018 and May 2021. Nine patients with COVID-19-associated PE were included in the analysis and age–sex-matched to 27 PE unexposed patients (without COVID-19).

The demographic and baseline characteristics of the study population are summarized in Table 1. Of 36 patients, 24 (67%) were men and the mean age was 67 (SD 13.9) years. All patients were of European origins. One patient per group was on oral anticoagulation for other indications before the onset of PE.

Table 2 summarizes the main signs and symptoms recorded upon clinical presentation. Dyspnea was the most common symptom in both groups; tachycardia was present in 44% of patients with COVID-19 and in 37% of unexposed patients. A positive troponin was observed in 89% and 93% of patients with and without COVID-19, respectively, whereas the mean RV/LV ratio was 1.50 (SD 0.36) and 1.27 (SD 0.11), respectively. The vast majority of patients had a PE located in the main pulmonary arteries (100% of those with COVID-19 and 96.3% of unexposed patients), which was bilateral in all cases but for two patients from the non-COVID-19 group. All patients had a simplified Pulmonary Embolism Severity Index (sPESI) ≥ 1, and approximately 20% of patients in both groups were hemodynamically unstable. Patients tested positive for SARS-CoV2 at a median of 9 (Q1–Q3: 4–19) days before the diagnosis of acute PE, and a concomitant pneumonia was present in six of nine patients with COVID-19.

A higher proportion of unexposed patients needed intensive care unit support compared with COVID-19 patients (before USAT: 22% vs. 11%; after USAT: 44% vs. 18%). The mean duration of hospitalization at our institution was 6 (SD 4) days in patients with COVID-19 and 5 (SD 4) days in unexposed patients. Five of nine patients with COVID-19 had been previously hospitalized at another institution.

A summary of the type and dose of the antithrombotic therapies is available in Table S1: antithrombotic and thrombolysis regimens during hospitalization or at discharge. The median dose of alteplase used during USAT was 20 mg in both groups (*p*-value: 0.2; Mann–Whitney U test). For each patient, a time-in-therapeutic-range for UFH was calculated with interpolated Rosendaal’s method: COVID-19 patients spent a median of 46% of time in the UFH therapeutic range during 15-h lysis, whereas this percentage was 43% among patients without COVID-19 (*p*-value: 0.91; Mann–Whitney U test).

### 3.2. Early Hemodynamic Parameters

**Figure 1:**
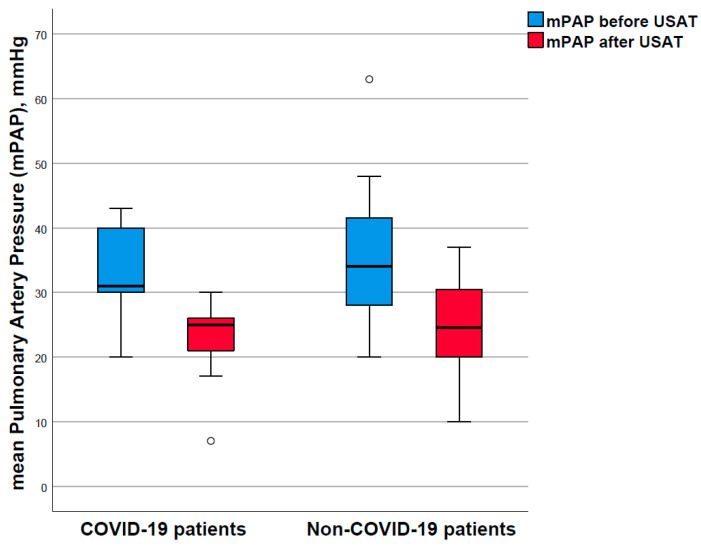
Invasively obtained mean pulmonary artery pressure before and after ultrasound-assisted catheter-directed thrombolysis (USAT). Patients with COVID-19-associated acute PE were age–sex-matched to patients without COVID-19 (1:3 ratio). Changes in the mean pulmonary arterial pressure (mPAP) before the treatment with ultrasound-assisted catheter-directed thrombolysis (USAT) and after the USAT: mPAP are depicted as median (Q1–Q3). Dots indicate outliers. Thicker lines indicate median values.

Among COVID-19 patients, invasively obtained mPAP decreased from a mean of 32.3 (SD 8.3) mmHg to 22.4 (SD 7.0) mmHg for a mean absolute difference of 9.9 (SD 6.2) mmHg (*p*-value = 0.001; paired t-test). Among unexposed patients, mPAP decreased from a mean of 35.4 (SD 9.7) mmHg to 24.6 (SD 7.0) mmHg for an absolute risk difference of 10.6 (SD 9.6) mmHg (*p*-value < 0.001; paired *t*-test); Figure 1 and Table S2: Invasive hemodynamic measurements. The mean initial mPAP value as well as the mean absolute mPAP reduction, and the difference of relative improvements, did not statistically differ between groups (*p* = 0.84, unpaired *t*-test).

**Figure 2:**
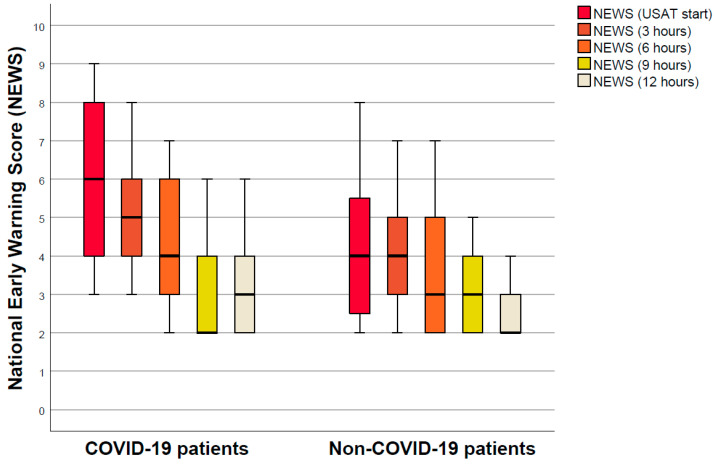
Course of the National Early Warning Score (NEWS). Changes in the National Early Warning Score (NEWS), depicted as median (Q1–Q3), during the first 12 h of treatment with catheter-directed lysis. Thicker lines indicate median values.

The NEWS decreased from a median of six (Q1–Q3: 4–8) points before USAT to three (Q1–Q3: 2–4) points after USAT among COVID-19 patients (*p*-value: 0.001; Wilcoxon signed-rank test). The NEWS decreased from a median of four (Q1–Q3: 2–6) points before USAT to two (Q1–Q3: 2–3) points after USAT among unexposed patients (*p*-value: 0.018; Wilcoxon signed-rank test); Figure 2. The percentage of improvement compared to baseline values was similar in patients with and without COVID-19 (*p* = 0.29, Mann–Whitney U test).

Consistently with the NEWS, heart rate, respiratory rate, and oxygen saturation showed progressive improvement over the first twelve hours from USAT start; Figure S1: Trend in vital parameters before and during the catheter directed lysis. Heart rate decreased from 96 (SD 15) bpm to 84 (SD 23) bpm after 12 h among patients with COVID-19 and from 90 (SD 15) bpm to 83 (SD 16) bpm among unexposed patients. The respiratory rate decreased from 24 (SD 6) to 18 (SD 3) per minute after 12 h among patients with COVID-19 and from 21 (SD 4) to 17 (SD 2) per minute among unexposed patients. The oxygen saturation increased from 94% (SD 2) to 96% (SD 2) after 12 h among patients with COVID-19 and from 96% (SD 2) to 97% (SD 2) among unexposed patients.

### 3.3. Clinical Events

No recurrent PE, major bleeding events, strokes, or device-related complications were observed in both groups within 30 days. One patient without COVID-19 had a recurrent PE event 6 weeks after the index acute PE.

Fourteen days after the diagnosis of acute PE, a patient with COVID-19 died because of COVID-19-related complications, which included pneumonia, severe sepsis, and multiorgan failure. No autopsy had been performed in this patient. No deaths were recorded among unexposed patients within 30 days of hospitalization and at long-term follow-up.

Among COVID-19 patients, two patients described residual respiratory symptoms at follow-up (dyspnea on exertion). All other patients reported a complete regression of symptoms. Echocardiographic data (Table S3: Echocardiographic data of COVID-19 patients at 3–6 months) were routinely collected only for patients with COVID-19 at long-term follow-up (month 3–6). The RV/LV ratio normalized in all patients. One COVID-19 patient resulted to be at an echocardiographic intermediate risk of pulmonary hypertension with increased pulmonary pressures on echocardiography; a complete CTEPH screening could have not been completed due to poor compliance and older age. No patient in either group had received a final diagnosis of CTEPH during 6-month follow-up.

## 4. Discussion

This study showed that USAT for COVID-19 associated acute PE with right ventricular dysfunction and severe embolic burden led to a rapid and clinically relevant reduction in pulmonary artery pressures, as well as an improvement of routine hemodynamic parameters. The magnitude of this reduction was similar between COVID-19 patients and age–sex-matched patients without COVID-19. Although it has been postulated that COVID-19 may trigger PE with peculiar pathophysiological mechanisms [19], USAT appears to be an effective therapeutic option for this subgroup of COVID-19 patients with centrally located acute PE associated with right ventricular dysfunction. This study represents the largest cohort study on the topic and confirms preliminary observations from isolated case reports [15,20,21].

Randomized controlled trials and prospective management studies showed that the improvement of echocardiographic parameters (RV/LV ratio) and thrombus burden among acute PE patients is rapid, usually within 24–48 h of USAT start [6,7,8]. These studies also proved the safety of this approach in terms of bleedings, particularly if compared to standard-dose systemic thrombolysis. This study showed that the degree of improvement of hemodynamic parameters among COVID-19 patients was similar to that of matched patients without COVID-19 and comparable to that described in studies from the literature. Moreover, as limited as this evidence can be, we could confirm prior safety data with no major bleedings recorded among 36 patients treated with the aforementioned regimen characterized by dose adjustments of heparin and t-PA according to standardized monitoring of anti-Xa activity and fibrinogen levels, respectively. The characteristics of the matched group population in terms of baseline hemodynamic impairment was very similar to that of prior interventional trials [6,7,8]. The ongoing Ultrasound-Facilitated, Catheter-Directed, Thrombolysis in Intermediate-High-Risk Pulmonary Embolism (HI-PEITHO) trial (NCT04790370) will demonstrate whether USAT improves clinical outcomes among an enriched population of intermediate-high acute PE patients compared with anticoagulation alone [22].

The present study also introduces other novelties. In particular, we described the time-depending changes of several clinical parameters that are routinely measured in real life during hospital stay at intermediate care unit. We showed that, consistently with individual parameters such as heart rate and oxygen saturation, the National Early Warning Score (NEWS) progressively improved in both groups during the first 12 h of USAT. In our study, the NEWS was initially higher among COVID-19 than in unexposed patients, possibly due to a double hit (PE and COVID-19 pneumonia), but it decreased to a similar level in both groups after USAT. Indeed, decisions on the frequency and type of reassessments of the hemodynamic status during the acute phase of PE are problematic in the absence of firm evidence. Of note, most current clinical risk models for acute PE rely on data collected at baseline, and the European guidelines cannot offer specific monitoring strategies. Moreover, the criteria to opt for an escalation of the reperfusion or supportive treatment (and timing) among apparently stable patients who are deteriorating necessarily remain based on individual experience. This is one of the first studies to investigate the NEWS among acute PE patients treated with reperfusion therapy and provides the first proof that it may be useful as a component of an efficacy outcome in the setting of interventional trials on thrombolytic therapies. Of note, the NEWS is a component of the primary efficacy outcome of the ongoing Ultrasound-Facilitated, Catheter-Directed, Thrombolysis in Intermediate-High-Risk Pulmonary Embolism (HI-PEITHO) trial (NCT04790370).

Finally, it has been suggested that thrombolysis may be effective among COVID-19 patients, particularly for those with Acute Respiratory Distress Syndrome, to restore the circulation and improve tissue oxygenation in smaller pulmonary vessels hit by so-called “immunothrombosis” [23,24,25,26]. In our study, the overall fatality among COVID-19 patients was low, especially if compared with those requiring intensive care unit stay from other cohorts. As limited as our experience may be, long-term echocardiographic data appeared reassuring. A recent pilot trial showed that low-dose systemic thrombolysis compared to anticoagulation alone did not seem to improve surrogate respiratory parameters among critically ill COVID-19 patients [27]. A number of interventional studies are ongoing to test whether different thrombolytic strategies may improve the survival and outcomes of COVID-19 patients with or without thromboembolic complications.

Our study has several limitations. The small number of patients limits the precision of risk estimates. Moreover, the retrospective and descriptive nature of the study represents an intrinsic barrier to its internal validity. The fact that our center is a leading institution for the development of novel reperfusion strategies for acute PE may explain why the rate of adverse events was very low: if implemented at other centers, the risks observed in this study may not apply. Finally, one cannot exclude the possibility that other supportive treatments may have contributed to the positive outcome of some patients.

In conclusion, we showed that mPAP rapidly decreased during USAT among patients with COVID-19-associated acute PE with a concomitant progressive improvement of hemodynamic and respiratory parameters, as measured by the NEWS. The magnitude of mPAP reduction was similar in patients with and without COVID-19.

## Figures and Tables

**Table 1:** Demographic and baseline characteristics.

	COVID-19 Patients (*n* = 9)	Non-COVID-19 Patients (*n* = 27)
Male sex, *n*/N (%)	6/9 (66.7)	18/27 (66.7)
Age (years), mean (standard deviation)	67.7 (13.9)	67.3 (13.8)
Body Mass Index (kg/m^2^), mean (standard deviation)	27.1 (3.8)	29.5 (6.9)
Active cancer, *n*/N (%)	1/9 (11)	4/27 (14.8)
Previous venous thromboembolism, *n* (%)	2/9 (22)	2/27 (7.4)
Surgery in the in the prior 30 days, *n* (%)	1/9 (11)	1/27 (3.7)
Immobilization in the prior 7 days, *n* (%)	1/9 (11)	2/27 (7.4)
Pregnancy or estroprogestinic therapy, *n* (%)	0/9 (0)	0/9 (0)
Dabigatran use, *n* (%)	1/9 (11)	0/27 (0)
Rivaroxaban use, *n* (%)	0/9 (0)	1/27 (3.7)
Aspirin use, *n* (%)	2/9 (22)	1/27 (3.7)
Coronary artery disease, *n* (%)	1/9 (11)	1/27 (3.7)
Renal insufficiency (CrCl < 50 mL/min), *n* (%)	1/9 (11)	3/27 (11.1)
Arterial hypertension, *n* (%)	3/9 (33.3)	12/27 (44.4)
Smoking, *n* (%)	0/9 (0)	4/13 (30.1)
Diabetes mellitus, *n* (%)	1/9 (11)	5/27 (18.5)
Chronic heart failure, *n* (%)	0/9 (0)	0/27 (0)

Patients with COVID-19-associated acute PE were age–sex-matched to patients without COVID-19 (1:3 ratio). *n* (number); N (total number).

**Table 2:** Clinical presentation and pulmonary embolism severity.

	COVID-19 Patients (*n* = 9)	Non-COVID-19 Patients (*n* = 27)
Dyspnea at rest, *n* (%)	7/9 (78)	25/27 (93)
Thoracic pain, *n* (%)	4/9 (44)	8/27 (29)
Cough, *n* (%)	4/9 (44)	6/27 (22)
Hemoptisis, *n* (%)	1/9 (11)	0/27 (0)
Syncope, *n* (%)	0/9 (0)	7/27 (26)
Temperature > 37.5 °C, *n* (%)	3/9 (33)	6/27 (22)
Tachycardia > 100 (beats/minute), *n*/N (%)	4/9 (44)	10/27 (37)
Heart rate (beats/minute), mean (SD)	96 (19)	90 (15)
Blood arterial pressure (mmHg), mean (SD)	124 (18)/63 (10)	134 (24)/68 (15)
Respiratory rate (acts/minute), mean (SD)	24 (6)	21 (4)
Oxygen saturation during oxygen supplement (%), mean (SD)	94 (2)	96 (2)
Troponin test positive, *n*/N (%)	8/9 (89)	25/27 (93)
Troponin I (ng/mL), mean (SD)	165 (145)	102 (88)
D-Dimer (ng/mL), median (Q1–Q3)	20 (6–20)	11 (6.3–30.3)
Fibrinogen (g/L), median (Q1–Q3)	4 (3–5)	4 (3–5)
NT-proBNP (ng/L), mean (SD)	4125 (5580)	4900 (6866)
RV/LV ratio by CT, mean (SD	1.5 (0.36)	1.27 (0.11)
Pulmonary embolism location, *n*	Central: 9/9 (100) Bilateral: 9/9 (100)	Central: 26/27 (96.3) Bilateral: 25/27 (92.6)
Intubation, *n* (%)	0/9 (0)	1/27 (3.7)
ESC high-risk class, *n* (%)	2/9 (22)	5/27 (19)
ESC intermediate-high-risk class, *n* (%)	7/9 (77)	22/27 (81)
Hospitalization time (days), mean (SD)	6 (4)	5 (4)

Patients with COVID-19-associated acute PE were age–sex-matched to patients without COVID-19 (1:3 ratio). Abbreviations: right ventricle/left ventricle (RV/LV) ratio; standard deviation (SD); N-terminal prohormone of brain natriuretic peptide (NT-proBNP); European Society of Cardiology (ESC); *n* (number); N (total number).

## Data Availability

Data are available upon request. Inquiries should be sent to stefano.barco@usz.ch.

## Supplementary Materials

The following supporting information can be downloaded at: https://www.mdpi.com/article/10.3390/v14081606/s1, Table S1: Antithrombotic and thrombolysis regimens during hospitalization or at discharge, Table S2: Invasive hemodynamic measurements, Table S3: Echocardiographic data of COVID-19 patients at 3–6 months, Figure S1: Trend in vital parameters before and during the catheter directed lysis.
