# Supplementary material for: Fixed-Dose Ultrasound-Assisted Catheter-Directed Thrombolysis for Acute Pulmonary Embolism Associated with COVID-19
Source: Viruses. 2022 Jul 22;14(8):1606. doi: 10.3390/v14081606 (PMC9394471; doi:10.3390/v14081606)
Supplement: Supplementary file 1 [file viruses-14-01606-s001.zip › viruses-1813347-supplementary.pdf]

Table S1. Antithrombotic and thrombolysis regimens during hospitalization or at discharge.

	**COVID-19 Patients (*n*=9)**	**Non-COVID-19 Patients (*n*=27)**
Initial therapeutic-dose unfractionated heparin (before USAT), *n* (%)	6/9 (66)	23/27 (85)
Therapeutic-dose unfractionated heparin during USAT, *n* (%)	9/9 (100)	27/27 (100)
Alteplase dose, median (Q1-Q3)	20 (20–20)	20 (10–20)
Mean anti-Factor Xa activity, mean (SD)	0.3 (0.7)	0.2 (0.7)
Time in therapeutic range (interpolated method, %), median (Q1-Q3)	46 (10–100)	43 (0–80)
Time to switch to direct oral anticoagulants or LMWH after catheter removal (days), median (Q1-Q3)	1.0 (1.0–6.5)	2.0 (1.0–2.0)
Therapeutic-dose LMWH, *n* (%)	3/9 (33)	0/27 (0)
Rivaroxaban, *n* (%)	4/9 (44)	18/27 (67)
Apixaban, *n* (%)	3/9 (33)	9/27 (33)
Edoxaban, *n* (%)	1/9 (11)	0/27 (0)

Patients with COVID-19-associated acute PE were age-sex matched to patients without COVID-19 (1:3 ratio). Abbreviation: ultrasound-assisted catheter-directed thrombolysis (USAT); low-molecular-weight heparin (LMWH).

Table S2. Invasive hemodynamic measurements.

	**COVID-19 Patients (*n*=9)**			**Non-COVID-19 Patients (*n*=27)**		
mmHg	*Before* USAT	*After* USAT	*Absolute difference*	*Before* USAT	*After* USAT	*Absolute difference*
**mPAP, mean (SD)**	32.3 (8.3)	22.4 (7.0)	9.9 (6.2)	35.4 (9.7)	24.6 (7.0)	10.6 (9.6)**
**sPAP, mean (SD)**	48.3 (9.7)	30.0 (10.0)	18.3 (9.9)*	51.6 (14.8)	33.2 (9.5)	17.9 (12.3)***
**dPAP, mean (SD)**	26.1 (5.9)	18.4 (6.1)	7.8 (5.9)*	26.5 (9.8)	18.7 (6.5)	7.9*** (9.9)

mPAP = mean pulmonary arterial pressure; sPAP = systolic pulmonary arterial pressure; dPAP= diastolic pulmonary arterial pressure; USAT = ultrasound-assisted catheter-directed thrombolysis.
**n*=1 missing; ***n*=3 missing, ****n*=5 missing.

Table S3. Echocardiographic data of COVID-19 patients at 3-6 months.

	**Baseline**	**Follow-up (3–6 Months)**
RV/LV Ratio, mean (SD)	1.13 (0.06)	0.93 (0.05)
TAPSE, mean (SD), mm	15.3 (3.2)	27.2 (6.7)
RV/RA pressure gradient, mean (SD), mmHg	n.a.	33.7 ± 11.1
Minimum IVC diameter, mean (SD), mm	24 (3.46)	n.a.
Pulmonary hypertension risk class, *n*	-	Low-risk: *n* = 6<br>Intermediate-risk: *n* = 1

Abbreviations: right ventricle/left ventricle (RV/LV) ratio; tricuspid annular plane systolic excursion (TAPSE), right atrial (RA); inferior vena cava (IVC); standard deviation (SD)

Figure S1. Trend in vital parameters before and during the catheter directed lysis.

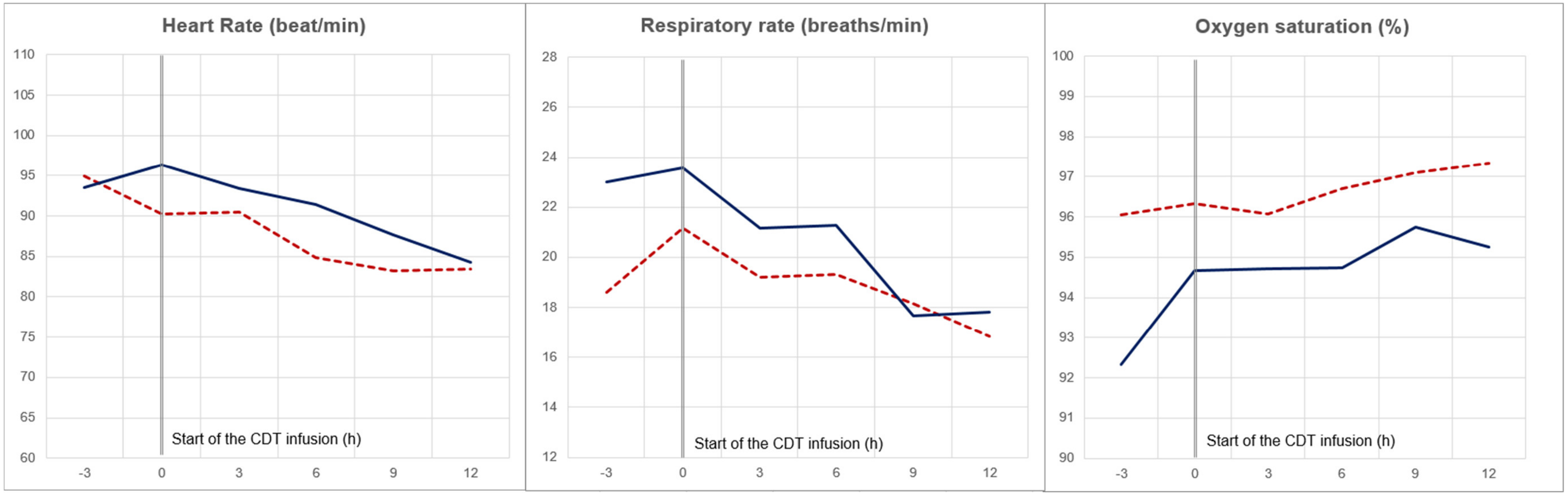

Changes in vital parameters from 3 hours before the start of the catheter directed lysis (hour -3 on the x axis) to 12 hours after lysis (hour 12 on the x axis). Values are expressed as means. Blue lines: patients with COVID-19. Red lines: patients without COVID-19.
